# Diffusion tensor imaging measurements for computer-aided diagnosis of autism: derived data from ABIDE II

**DOI:** 10.3389/fpsyt.2026.1819266

**Published:** 2026-09-10

**Authors:** Noel A. Cardenas-Hernandez, Marlen Perez-Diaz, Karla Batista García-Ramó, Maria del C. Valdés Hernández

**Affiliations:** 1 Universidad Central “Marta Abreu” de Las Villas, Department of Physics, Santa Clara, Cuba; 2 Universidad Central “Marta Abreu” de Las Villas, Department of Automatics, Santa Clara, Cuba; 3 Centre for Neuroscience Studies, Department of Medicine, Queen’s University, Kingston, ON, Canada; 4 Clinical Research Department, Center of Isotopes, Havana, Cuba; 5 Centre for Clinical Brain Sciences, Department of Neuroimaging Sciences, University of Edinburgh, Edinburgh, United Kingdom

**Keywords:** axial diffusivity, descriptive statistics, fractional anisotropy, image IDs, mean diffusivity, measurements, radial diffusivity

## Abstract

Advancing the understanding of neuroimaging biomarkers in neurodevelopmental research requires structured, well-documented datasets to support reproducibility and comparative analyses. This dataset comprises diffusion MRI-derived measurements from the Autism Brain Imaging Data Exchange (ABIDE II) database—fractional anisotropy (FA), axial diffusivity (AD), mean diffusivity (MD), and radial diffusivity (RD)—across multiple brain regions for both ASD and control groups, associated with unique image identifiers. Data were generated using standardized image acquisition and computational processing pipelines, resulting in uniformly processed and reproducible quantitative region-specific metrics to facilitate cross-sectional comparative studies. The dataset is structured with explicit metadata, detailed variable descriptions, and complete case records to support programmatic access and automated workflows. Data visualization and harmonization codes accompany the dataset. This resource enables the investigation of white matter microstructural properties in relation to autism through different analytical approaches, including hypothesis testing, machine learning, and meta-analyses. The structured format and transparent documentation promote its reuse in other neuroimaging studies. FAIR^2^Data Portal: https://www.doi.org/10.71728/senscience.dbrh-5zc8.

## 1 Introduction

Robust and reproducible data resources are fundamental to advancing the objective identification, characterization, and management of neurological and developmental disorders. In the context of neuroimaging and neurodevelopmental conditions such as autism spectrum disorder (ASD), transparent documentation of data structure, measurement methods, and associated metadata is critical for supporting secondary analyses and enabling integration with machine learning workflows. The detailed annotation and stewardship of such datasets are increasingly important as the research community seeks interoperable resources that support both traditional statistical approaches and modern artificial intelligence (AI)-driven analyses, while ensuring the transparent assessment of methodological limitations, variable definitions, and provenance.

The dataset provided in this data article comprises quantitative brain white matter measurements derived from the processing of diffusion tensor imaging (DTI) scans, with subject-level annotation of ASD or control group membership. Data originate from a subset of the publicly available Autism Brain Imaging Data Exchange (ABIDE II) repository (1), representing three acquisition sites and uniformly processed according to a documented imaging and postprocessing protocol (2). For each individual, spatially aggregated DTI features—including fractional anisotropy (FA), mean diffusivity (MD), axial diffusivity (AD), and radial diffusivity (RD)—are extracted within 25 white matter regions of interest, defined by the JHU ICBM-DTI-81 neuroanatomical atlas (3). Demographic variables such as image identifier and group designation anchor each record in the main ABIDE II database, supporting linkage to the raw neuroimaging files, principal metadata, and supplementary clinical information from this database.

Results from processing the neuroimaging data from the ABIDE II database, also available to the scientific community, include the Preprocessed Connectome Project (PCP; http://preprocessed-connectomes-project.org/abide/), which uses functional and structural magnetic resonance imaging (MRI) data, and an additional dataset with the output from the modification of a regularized dimensionality reduction algorithm, namely modified locally linear embedding (MLLE), which contains the quantum fluctuations as measured by functional MRI (fMRI) (https://fcon_1000.projects.nitrc.org/indi/abide/LLE_home.html). The Large-Scale Curated Diffusion MRI Data for Autism Research (LADDER) project (https://www.nitrc.org/projects/abide2-dwi-bids/) organizes the DTI data from ABIDE II according to the Brain Imaging Data Structure (BIDS; 4), but there is a lack of ready-available diffusion parameters for these data. Researchers wanting to use the DTI data from this rich and well-characterized dataset need to have the computational resources, skills, and time to process the images and then check and validate the processing results, which may not always be possible within available project timeframes and funding or with the computational resources available at educational institutions in developing countries. These limitations could seriously affect and bias autism research and the usability of the DTI data from ABIDE II.

This dataset provides spatially resolved valid neuroimaging-derived features (i.e., tabulated values in comma-separated files) for 150 subjects (87 with ASD, 63 controls) across a consistent set of anatomically labeled regions, accompanied by statistics that allow assessment of central tendency, dispersion, and data completeness. Data structure and codebook entries adhere to schema.org and MLCommons Croissant v1.0 conventions to facilitate AI-readiness, interoperability, and reuse. All feature construction and processing steps—including segmentation, diffusion parameter calculation, and region-level aggregation—are documented and reproducible via the software and method described in Cárdenas-Hernández et al. (2). Additionally, information on acquisition site, image identifiers, and preprocessing pipeline ensures traceability and methodological transparency.

By providing a rigorously described dataset under findable, accessible, interoperable, reusable, AI-readiness, and responsible AI (FAIR^2^) principles, this resource is designed to support hypothesis-driven research, benchmarking of classification algorithms, and the development of responsible AI applications in neuroimaging for ASD. Through the explicit definition of data provenance, processing, and variable semantics, the dataset aims to facilitate both secondary data analyses and method development in the neuroscience and machine learning communities, while supporting ethical reuse and compliance with emerging standards for responsible AI in biomedical research.

## 2 Methods summary

### 2.1 Study design

This dataset is constructed from neuroimaging and demographic data obtained through the ABIDE II open-access repository (1), focusing on structural and diffusion tensor brain features associated with ASD. Subject selection was based on the availability of complete DTI data, specifically requiring both b-value (bval) and b-vector (bvec) files essential for subsequent diffusion analysis. Only subjects from three designated research centers—New York University Langone Medical Centre (NYU) (consisting of two samples acquired with different scanning parameters), San Diego State University (SDSU), and Trinity Centre for Health Sciences (TCD)—were included, as each provided the required imaging series in addition to the comprehensive metadata common to all contributing centers to the ABIDE II database, thereby supporting reproducible analytic work. Inclusion was limited to individuals possessing both DTI and T1-weighted anatomical MRI scans, along with all mandatory diffusion parameter files. Subjects missing any critical imaging component or presenting with incomplete or compromised images were excluded to enforce protocol homogeneity. The resulting cohort was stratified by diagnostic annotation from the ABIDE II resource, yielding 87 ASD and 63 control individuals with valid data. For each subject, demographic variables comprising age, sex, and handedness are recorded in the ABIDE II database to inform later modeling and assess cohort balance via linkage with the present dataset through the image_id field.

### 2.2 MRI data acquisition

As previously mentioned, imaging data were acquired at NYU, SDSU, and TCD, each utilizing a 3-T MRI system with site-specific hardware: SIEMENS MAGNETOM Allegra at NYU, GE 3T MR750 at SDSU, and Philips Intera Achieva at TCD. MRI acquisition protocols for DTI differed by center, with NYU’s samples employing echo times (TE) and repetition times (TR) of 78/5,200 ms and 3.25/2,530 ms, respectively, and SDSU/TR = min/8,500 ms, while TCD used TE/TR = 79/20,244 ms. The NYU series included 64 diffusion directions at differing voxel resolutions (3.0 mm^3^ × 3.0 mm^3^ × 3.0 mm^3^ and 1.3 mm^3^ × 1.0 mm^3^ × 1.3 mm^3^), and both SDSU and TCD datasets provided 61 directions at 2.0 mm^3^ × 2.0 mm^3^ × 2.0 mm^3^. All source images, coupled with associated bval and bvec files, underwent integrity checks to ensure the presence and compatibility of each component prior to analysis. This procedure systematically excluded file sets with missing elements, maintaining a consistent and reproducible starting point for subsequent steps.

### 2.3 Image preprocessing and feature extraction

Standardized preprocessing was applied to enhance image quality and analytical validity across the multicenter sample. DTI series were subjected to denoising and corrections for artifacts such as Gibbs ringing, susceptibility distortions, and head motion, employing MRtrix3 (5) and FMRIB Software Library (FSL) software (https://fsl.fmrib.ox.ac.uk/fsl/docs/). Intensity inhomogeneity was adjusted through bias field correction, while normalization was conducted to reduce inter-scan variability. For scans lacking an unweighted diffusion image with reverse phase-encoding, synthetic b = 0 images were generated using Synb0-DISCO (6), integrating T1-weighted anatomical data to facilitate proper geometric correction.

Voxelwise diffusion maps were computed using FSL’s diffusion tools. Four principal scalar metrics were derived: fractional anisotropy (FA), mean diffusivity (MD), axial diffusivity (AD), and radial diffusivity (RD). FA was calculated as:


$$\text{FA}=\;\sqrt{\frac{1}{2}(\frac{{\left(\lambda_{1}-\lambda_{2}\right)}^{2}+{\left(\lambda_{2}-\lambda_{3}\right)}^{2}+{\left(\lambda_{3}-\lambda_{1}\right)}^{2}}{{\lambda_{1}}^{2}+{\lambda_{2}}^{2}+{\lambda_{3}}^{2}})}$$

which is equivalent to:


$$\text{FA}=\;\sqrt{\frac{3}{2}(\frac{{\left(\lambda_{1}-\bar{\lambda }\right)}^{2}+{\left(\lambda_{2}-\bar{\lambda }\right)}^{2}+{\left(\lambda_{3}-\bar{\lambda }\right)}^{2}}{{\lambda_{1}}^{2}+{\lambda_{2}}^{2}+{\lambda_{3}}^{2}})}$$

MD represented the mean of the three eigenvalues of the diffusion tensor:


$$\text{MD}=\frac{\lambda_{1}+\lambda_{2}+\lambda_{3}}{3}$$

AD corresponded to the principal eigenvalue ( $\text{AD}=\lambda_{1}$), while RD was defined as $\text{RD}=(\lambda_{2}+\lambda_{3})/2$.

Processed metric maps for each subject were spatially aligned to the JHU ICBM-DTI-81 white matter atlas (3) using affine registration via FSL’s flirt function (7), establishing consistent anatomical localization of features. For each atlas-defined region, regional averages were computed by aggregating voxelwise metric values, thereby generating a multidimensional feature vector per image. These vectors were organized into tabular comma-separated value (CSV) files, separately for ASD and control subjects, and included field names reflecting metric and region codes.

### 2.4 Definition of white matter subregions for feature extraction

Region selection was informed by previous literature linking specific white matter tracts to ASD traits. From the full set of 48 atlas-defined subregions, 25 regions were prioritized to focus analyses on tracts with reported ASD associations. These included subdivisions of the corpus callosum, the cerebral peduncle, the anterior and posterior internal capsule, the retrolenticular internal capsule, the anterior and superior corona radiata, the inferior longitudinal fasciculus, the external capsule, the cingulum, and the superior longitudinal fasciculus. For each subject and region, FA, MD, AD, and RD were spatially averaged, yielding a total of 100 regional features per scan. File naming conventions and region codes were strictly maintained to facilitate clear mapping between metrics and anatomical loci.

### 2.5 Dataset visualization and summary

A planned workflow for dataset visualization and summary reporting was established to promote interpretability and transparency. For each DTI metric and white matter region, visualizations such as histograms and boxplots would be used to illustrate feature distributions, enabling direct visualization of dispersion, outliers, and range. Additional comparative plots—such as grouped boxplots or violin plots—were specified to enable assessment of potential group-level differences between ASD and control samples within each tract, supporting examination of within- and between-group overlap. Complementary summary tables would present descriptive statistics (mean, median, standard deviation, minimum, and maximum) for each feature, stratified by cohort. Employing both graphical and tabular approaches facilitates user understanding of variability and group-specific trends, which is essential for downstream studies and secondary analyses.

### 2.6 Dataset finalization and output preparation

Final dataset structuring involved careful assembly, validation, and formatting of all extracted DTI-derived features into master CSV files for ASD and control groups. Each record corresponded to a unique subject and contained comprehensive regional feature vectors, labeled according to established conventions and including an explicit image identifier for provenance tracking. We provide subject IDs for each set of data, which can be linked to the main ABIDE II database for further specific analyses. Both automated and manual verification ensured all required variables and fields were accurately present and that their naming was harmonized with ABIDE II and atlas documentation. Any anomalies or missing values were identified and addressed through flagging or exclusion. For each field, descriptive statistics were calculated to provide users with quantitative benchmarks for completeness and distribution, contributing to quality control and transparency. Data preparation culminated in a dataset configuration optimized for reuse, reproducibility, and objective evaluation in further research.

## 3 Data overview

### 3.1 Data summary

The dataset consists of regional DTI metrics extracted from the ABIDE II open-access neuroimaging repository, comprising 87 individuals with ASD and 64 typical control subjects across three contributing centers. For one of the typical controls (image_id 29234), the standard processing pipeline failed, yielding valid data for 63 controls. For each subject, scalar diffusion metrics—including FA, AD, MD, and RD—were computed following a standardized preprocessing pipeline and projection to anatomically defined ASD-relevant white matter regions.

Data fields reflect spatially averaged DTI measures within 25 white matter subregions, selected *a priori* from the JHU ICBM-DTI-81 White Matter Atlas based on relevance to ASD as indicated in the literature. Feature matrices present data in tabular CSV format, with each row representing an individual subject and columns encoding diffusion metrics by region (e.g., fa_3, ad_3, md_3, rd_3 for region 3). All regional mean values derive from voxelwise calculations using validated FSL and MRtrix3 routines, ensuring cross-subject comparability. Data completeness was confirmed through automated and manual quality checks, excluding records with missing or invalid imaging or demographic metadata.

Descriptive statistics for each feature—such as mean, standard deviation, minimum, and maximum values—are provided to enable downstream assessment of feature distributions across groups and regions. The accompanying metadata establish the provenance, anatomical mapping, and methodological lineage of each variable. This dataset structure supports analytic reproducibility, machine learning reuse, and integration with broader neuroimaging resources, while retaining alignment with contemporary interoperability and FAIR^2^ data stewardship standards.

### 3.2 Quantitative summary of the dataset

#### 3.2.1 Total number of records

The dataset comprises a total of 151 records corresponding to individual neuroimaging subjects. These records are split into two main cohorts: 87 records for subjects diagnosed with ASD and 64 records for control subjects (one of which contains null data). Each record includes a set of quantified measurements across multiple white matter brain regions and DTI metrics. Every subject-level entry is uniquely identified by an image identifier (image_id), ensuring traceability to the original imaging data and group allocation.

#### 3.2.2 Coverage

Coverage in this dataset is defined by group status (ASD vs. control), region of interest, and DTI-derived metric. There are 25 unique white matter regions, as defined by the JHU ICBM-DTI-81 atlas, included for each subject. Within each region, four DTI-derived metrics are recorded: FA, AD, MD, and RD. This results in a feature vector of 100 region-metric combinations per subject. The dataset structure ensures that each of these 100 features is present for all 151 subject records, with no missing values and only one control subject’s invalid data reported at the field level.

#### 3.2.3 Attribute diversity

Each record contains 100 distinct quantitative features—one for each region-metric pairing—structured consistently across all subjects. Regions cover cerebral tracts such as the corpus callosum, cerebral peduncle, internal capsule subdivisions, corona radiata, inferior longitudinal fasciculus, external capsule, cingulum, and superior longitudinal fasciculus (see Table 2 in 2). The attribute set reflects diversity in both neuroanatomical coverage and DTI measurement type, supporting multifactorial analyses across biological structures and imaging modalities.

#### 3.2.4 Quantitative attributes

The principal quantitative variables represented in the dataset are DTI metrics: FA, AD, MD, and RD, calculated for each of 25 white matter regions per subject. Each metric exhibits variation both within and across cohorts. For example, in region 3 (corpus callosum), FA values in the ASD group (*n* = 87) range from 0.339 to 0.639 (mean: 0.525, standard deviation [SD]: 0.057), while in the control group (*n* = 64), values range from 0.0 to 0.672 (mean: 0.541, standard deviation: 0.084). AD values (mm^2^/s) for the same region range from 0.00122 to 0.00191 (mean: 0.00155, SD: 0.00014) for ASD and from 0.0 to 0.00181 (mean: 0.00150, SD: 0.00023) for controls. It is worth pointing out that these values in the control subgroup are affected by the presence of void data from a control subject. Across all features, the mean, minimum, maximum, and standard deviation are documented, and descriptive statistics are similarly available for each region and DTI measure in both groups. Statistical comparisons of FA and MD values between the ASD and control groups in each region, as well as tests demonstrating high collinearity between MD, AD, and RD across all regions in both population groups, were conducted and published previously (2). This pattern of reporting is consistent across all 25 included regions. The variability of measurements reflects underlying biological and group-related heterogeneity, while field-level statistics support transparency and secondary analysis.

#### 3.2.5 Data completeness

The dataset achieves full completeness for all included features across both the ASD and control groups. Each of the 100 regional-metric variables (for a total of 15,100 values—of which 15,000 are valid—across all subjects) is populated with quantitative measurements, and no missing values are present for any field. This integrity is corroborated by automated verification procedures and summary statistics, allowing reproducible, unbiased secondary analyses.

### 3.3 Dataset availability

The dataset is FAIR^2^-certified and publicly available under the Open Data Commons Attribution License (ODC-By v1.0), permitting unrestricted reuse with appropriate attribution. Access is provided via (1) the FAIR^2^ Data Portal (https://doi.org/10.71728/senscience.dbrh-5zc8), supporting interactive exploration and quality assessment, and (2) the downloadable FAIR^2^ Data Package containing raw and processed data, structured metadata, scripts, and detailed methods. The dataset is archived with a persistent identifier and integrated into the relevant neuroimaging data ecosystem. All experimental and processing procedures are fully documented. Licensing terms and data use conditions are specified in the accompanying documentation. FAIR^2^ Data Package DOI: https://doi.org/10.71728/senscience.dbrh-5zc8.

### 3.4 FAIR^2^ compliance introduction

**Table 1** presents an itemized assessment of the dataset against the FAIR^2^ framework. It summarizes adherence to each principle, documenting the presence of persistent identifiers, metadata standards, interoperability practices, accessibility protocols, and measures to support ethical AI use and transparency. Each criterion is evaluated and accompanied by practical evidence, implementation notes, or links to protocol documentation. Furthermore, **Table 1** is designed to facilitate the independent verification of dataset compliance with current FAIR^2^ requirements, enabling users to quickly ascertain the strengths and limitations of the dataset’s stewardship, technical accessibility, and ethical readiness for AI applications. This overview supports transparent communication to both human researchers and automated systems regarding the dataset’s structure, provenance, licensing, and suitability for responsible reuse and integration into computational workflows.

**Table 1:** The FAIR^2^ Compliance Certification presented here was generated through a Human-in-the-Loop (HITL) process combining automated FAIR^2^ system checks with author-supplied inputs. While certain metadata fields and validations (e.g., DOI registration, schema adherence, file accessibility) are verified automatically by the FAIR^2^ platform, other elements—such as domain-specific documentation quality and Responsible AI considerations—reflect expert curation by the dataset authors.

Criteria	Details	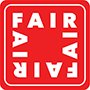
Findability (F)		
Unique identifier	The dataset is referenced with persistent identifiers corresponding to the ABIDE II repository and distinct output CSV file names supporting unambiguous citation, tracking, and reuse. Inclusion in future DOI-registered repositories will further enhance its global traceability.	
Metadata	Metadata include the dataset title, responsible research groups, subject group (ASD, control), image/subject IDs, contributing institutions, MRI acquisition details (scanner model, field strength, acquisition protocols), region codes/names (JHU ICBM-DTI-81), field definitions (FA, AD, MD, RD), and basic subject characteristics. Temporal and geographic coverage are specified by site and scan cohort. Additional metadata, such as measurement uncertainty and sample processing logs, could further support usability.	
Metadata includes data identifiers	All distributed metadata and tabular data files include image_id fields linking records to the originating repositories and raw ABIDE II IDs, ensuring an explicit and persistent association between metadata and dataset contents.	
Searchable metadata	The use of region codes (from the JHU ICBM-DTI-81 atlas) and explicit variable conventions promotes metadata consistency.	
Indexed in repositories	The source ABIDE II database is indexed in established neuroinformatics repositories. The dataset is structured for further indexing in the FAIR^2^ Data Portal and general-purpose open repositories. Expanding the listing to additional data portals could further enhance domain-spanning discoverability.	
Accessibility (A)		
Open access	The dataset is derived exclusively from publicly accessible sources (ABIDE II) and is structured for unrestricted access under standard research data policies. No registration or fee is required.	
Long-term access	Archival is supported by hosting in ABIDE II along with structured output files, which can be deposited in institutional and community repositories with multisite backups. Mirrored redundancy or cloud-mirroring strategies may further secure long-term availability.	
API access	The ABIDE II repository provides batch access, and the outputs (CSV files) are directly downloadable. Interactive visualizations, descriptive summary tables, and API endpoints can be added as part of future FAIR^2^-enhanced dissemination.	
Interoperability (I)		
Standardized formats	All data are provided as comma-separated value (CSV) files, compatible with common statistical analysis and machine learning pipelines. Imaging references adhere to NIfTI-1 standards. Output file field naming aligns with atlas region codes and measurement conventions. Croissant metadata (MLCommons) and schema.org descriptors are available for advanced AI integration.	
Controlled vocabularies	Region names and codes are standardized via the JHU ICBM-DTI-81 atlas. Quantitative imaging metrics use established neuroimaging terminology (FA, AD, MD, RD). Full ontology annotation and mapping (e.g., NeuroLex, OBIB) will further enhance semantic interoperability.	
Cross-platform integration	Dataset structure and documentation follow best practices in neuroinformatics, supporting interoperability with neuroimaging analysis software (FSL, MRtrix3), open science platforms, and AI tools. Output conventions facilitate integration into clinical research, machine learning, and data harmonization workflows.	
Reusability (R)		
Comprehensive and rich documentation	Documentation includes sample inclusion/exclusion criteria, MRI and DTI pre-processing details, computation formulas for all derived metrics, region mapping, data provenance (center, scanner, software versions), and descriptive statistics for all variables. Each field and region is described with origin, calculation steps, and statistical descriptors.	
License	The dataset is released for open reuse under licensing consistent with the source data (ODC-BY or equivalent), permitting modification and redistribution with required attribution. All reusers are directed to credit the ABIDE II initiative and this dataset’s authorship.	
Detailed provenance	Metadata and process documentation explicitly track data from initial collection (contributing institutions and MRI protocols) through preprocessing (software/toolchain), feature extraction, and tabular output mapping. Each image_id is linked to participant records, acquisition site, and scan parameters.	
Domain-relevant standards	The dataset conforms to major neuroimaging data standards supporting seamless adoption in biomedical research. Further formal validation against BIDS for tabular outputs will strengthen compliance.	
Versioning and updates	Dataset updates and corrections are tracked through file versioning and changelogs. Previous versions are preserved for transparency, enhancing trust and reproducibility. Release notes detail changes in subject inclusion, processing pipelines, or variable definitions.	
AI-readiness (AIR)		
Structured for machine learning	The dataset is delivered in structured CSV files with explicit group labels (ASD/control), column-level documentation, and region–feature mapping compatible with supervised and unsupervised learning frameworks. Variables are labeled with clear region and metric identifiers.	
Scalable	The tabular design enables efficient handling in high-throughput computing and cloud-based analytics environments. File and variable conventions are compatible with distributed processing and scalable AI workflows.	
Training and validation sets	The ASD and control group outputs can be partitioned into training, validation, and test subsets as required by downstream ML protocols. Each record’s provenance supports stratified sampling and bias assessment during machine learning.	
Responsible AI (RAI)		
Ethical standards and misuse	The dataset is provided for research on neurodevelopmental and imaging-based analysis. It is not intended for direct clinical diagnosis, uncontextualized individual prognostics, or use outside the population/sample scope (pediatric/young adult ASD and controls). Documentation specifies intended application domains and methodological boundaries.	
Biases in the dataset	Geographic and demographic biases exist, with all records originating from three collection centers, and demographic details captured at inclusion. Disparities in group sizes, center protocols, or scanner types may affect model generalizability; such factors are extensively documented for bias assessment and mitigation during use.	
Data privacy and security	The dataset contains no direct personal identifiers. All individual records are anonymized according to ABIDE II policy. Data integrity is ensured via file checksums and alignment with the original repository. No further privacy risks are identified under current data use practices.	
Fairness and nondiscrimination	ABIDE II documentation provides transparent recording of sex, age, and handedness for each subject. Users are directed to assess fairness and potential bias, especially when deploying models in broader or clinical populations. Recommendations are provided regarding representative sampling and subgroup analysis.	
Explainability and interpretability	Each feature and field is fully defined, with region codes mapped to anatomical names and the methodology for metric computation explicitly stated. Data transformations and quality controls are described to support traceable and interpretable AI workflows.	
Ethical and social impact	The dataset is relevant to research in autism spectrum disorder and neurodevelopment, with potential translational value in identifying biomarkers. Ethical documentation cautions against over-extrapolation, improper clinical inference, and unsupported precision health claims based on this dataset alone.	
Human-in-the-loop (HITL) considerations	Manual quality control and preprocessing intervention are described as part of MRI data curation. Users reusing or deploying HITL systems are advised to ensure additional review, especially in quality assurance and outlier detection/interpretation.	
Participatory data	Data provenance tracks institutional and collaborative contributions, with full attribution of all contributing sites and processing steps. Community feedback and collaborative stewardship are encouraged in future dataset releases.	
AI safety and fairness	Potential AI risks, including overfitting to specific cohorts or overinterpretation of subtle group effects, are noted. Users are provided with guidelines for external validation and are recommended to transparently report model errors, uncertainties, and fairness metrics.	
Inclusion	The demographic composition (age, sex, center, handedness) is captured for all records in the ABIDE II database, supporting analyses for inclusivity and subgroup fairness. Stratified statistics can be generated to examine and correct for demographic imbalances. A direct link between this dataset and the ABIDE II database can be established through the image_id field.	
Annotation platforms and processes	Automated and manual checks are specified for feature extraction, data curation, and variable verification. All annotation protocols (imaging processing, subject labeling) are systematically documented for reliability and replicability.	
Lifecycle documentation	All stages from original collection, MRI/DTI preprocessing, feature extraction, quality control, output mapping, and downstream summary statistics are described in the methods and provenance records. Each step is referenced with the software, atlas, and parameter settings.	
Record-level metadata	Every record includes an image_id, which is consistent with center/source annotation, group status, and associated demographic fields in the ABIDE II database. This enables record-level documentation of heterogeneity and supports downstream safety/fairness evaluations.	
Overall FAIR^2^ Badge compliance	Compliant—the dataset qualifies for the FAIR^2^ Badge, meeting all necessary criteria. File structure, metadata, provenance, documentation, and openness enable reusability, AI integration, and responsible application. Minor future improvements could include formal DOI assignment, enrichment of semantic metadata, and further expansion of participatory and social impact documentation.	

### 3.5 Visual overview

**Figure 1:**
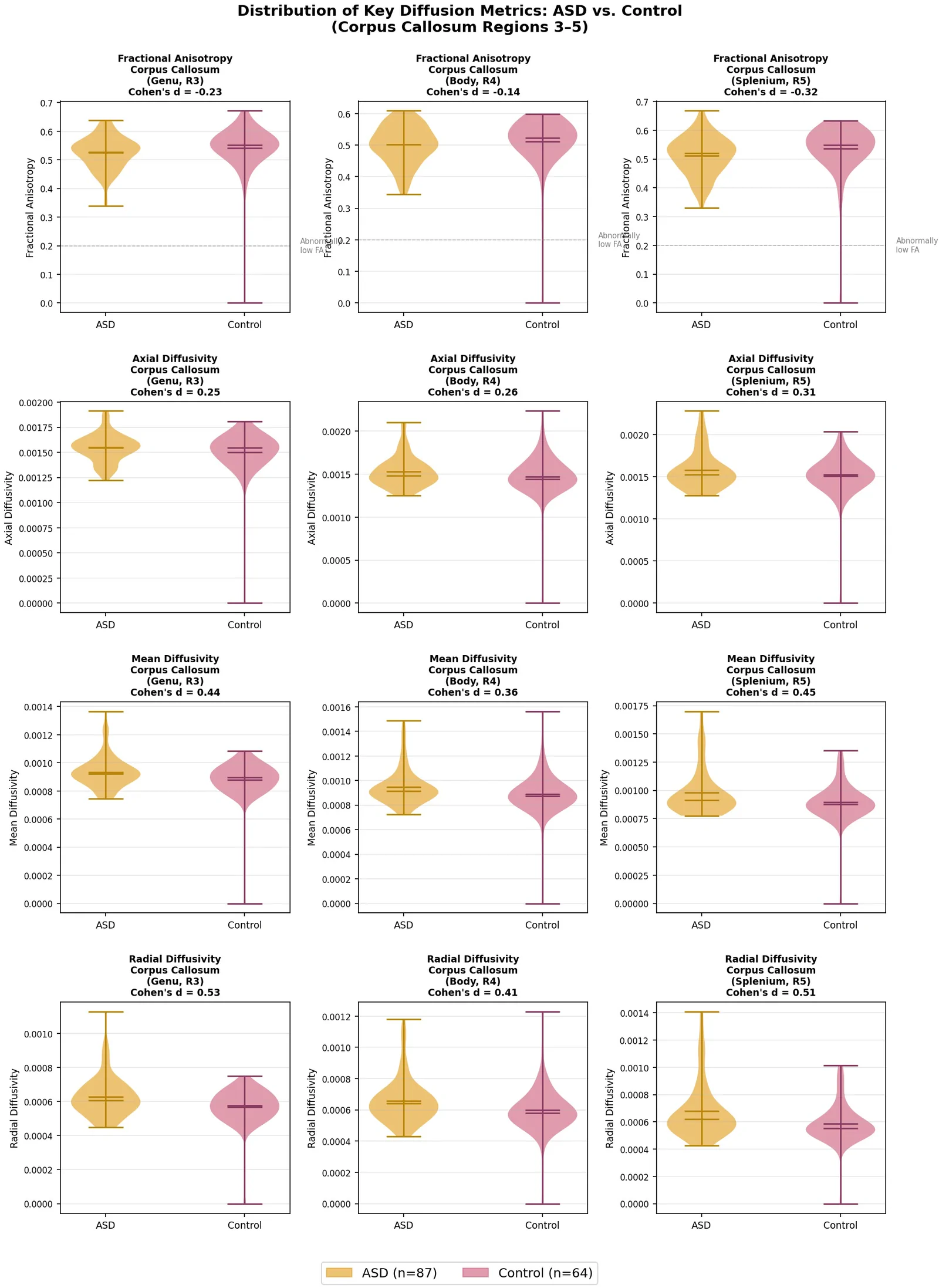
Distribution of key diffusion metrics: ASD vs. control (Corpus Callosum Regions 3–5). Group-wise distribution of fractional anisotropy (FA), axial diffusivity (AD), mean diffusivity (MD), and radial diffusivity (RD) in regions 3, 4, and 5 of the corpus callosum for individuals with autism spectrum disorder (ASD) and neurotypical controls. Violin plots summarize tract-level diffusion properties, with effect sizes (Cohen’s *d*) annotated for each comparison. Abnormally low FA values below a threshold in the control group involve a subject with void data (image_id 29234), highlighting the importance of data integrity and reporting, as well as group differences and spatial variation in major commissural white matter.

**Figure 2:**
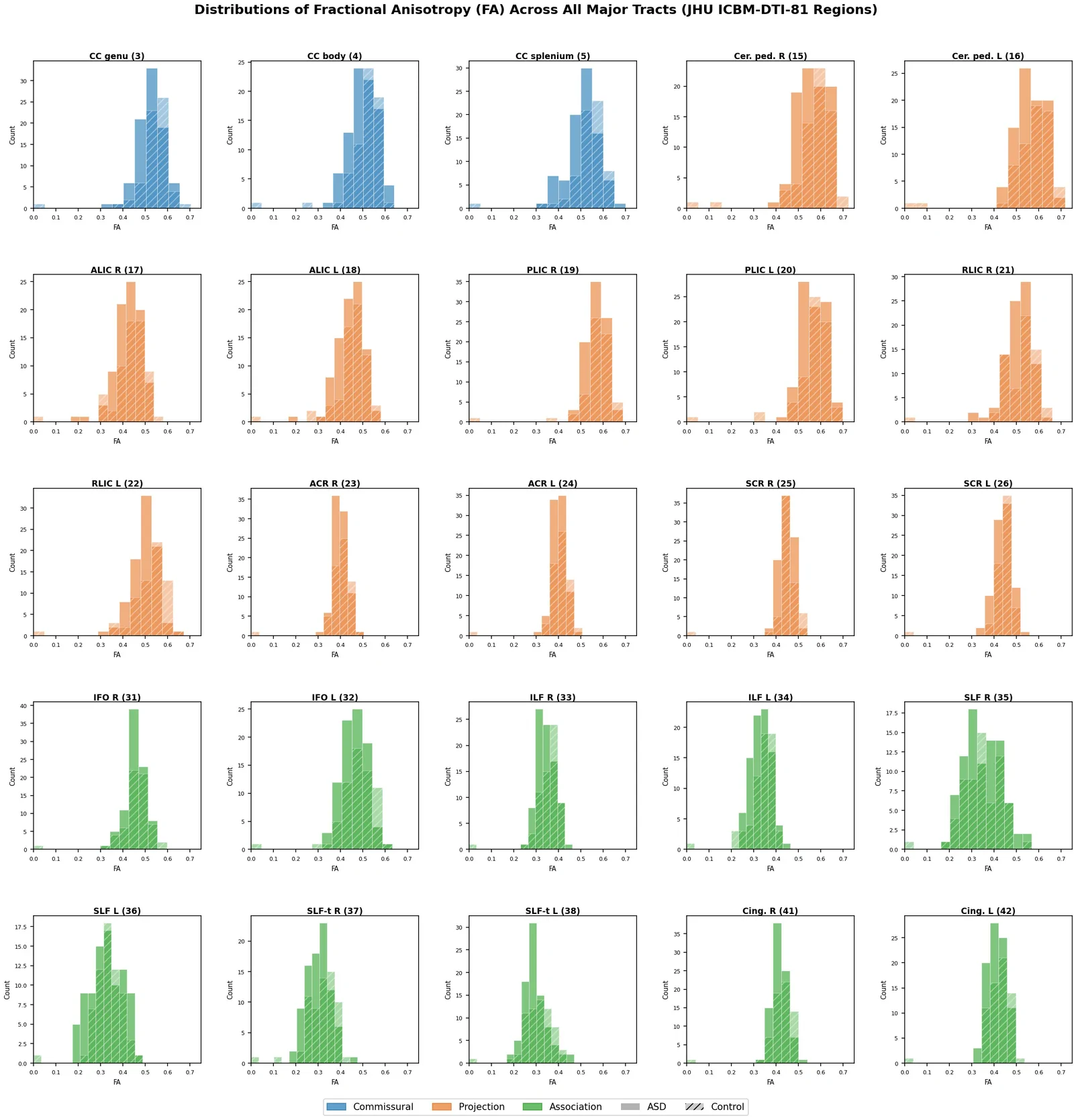
Distributions of FA across all major tracts (JHU regions). Distribution of fractional anisotropy (FA) values across major white matter regions in ASD and control cohorts, faceted by anatomical region and color-coded by tract system (commissural, projection, association). This visualization highlights interregional differences in FA, reveals outlier profiles, and identifies regions of high and low anisotropy relevant to white matter microstructure and clinical interest.

**Figure 3:**
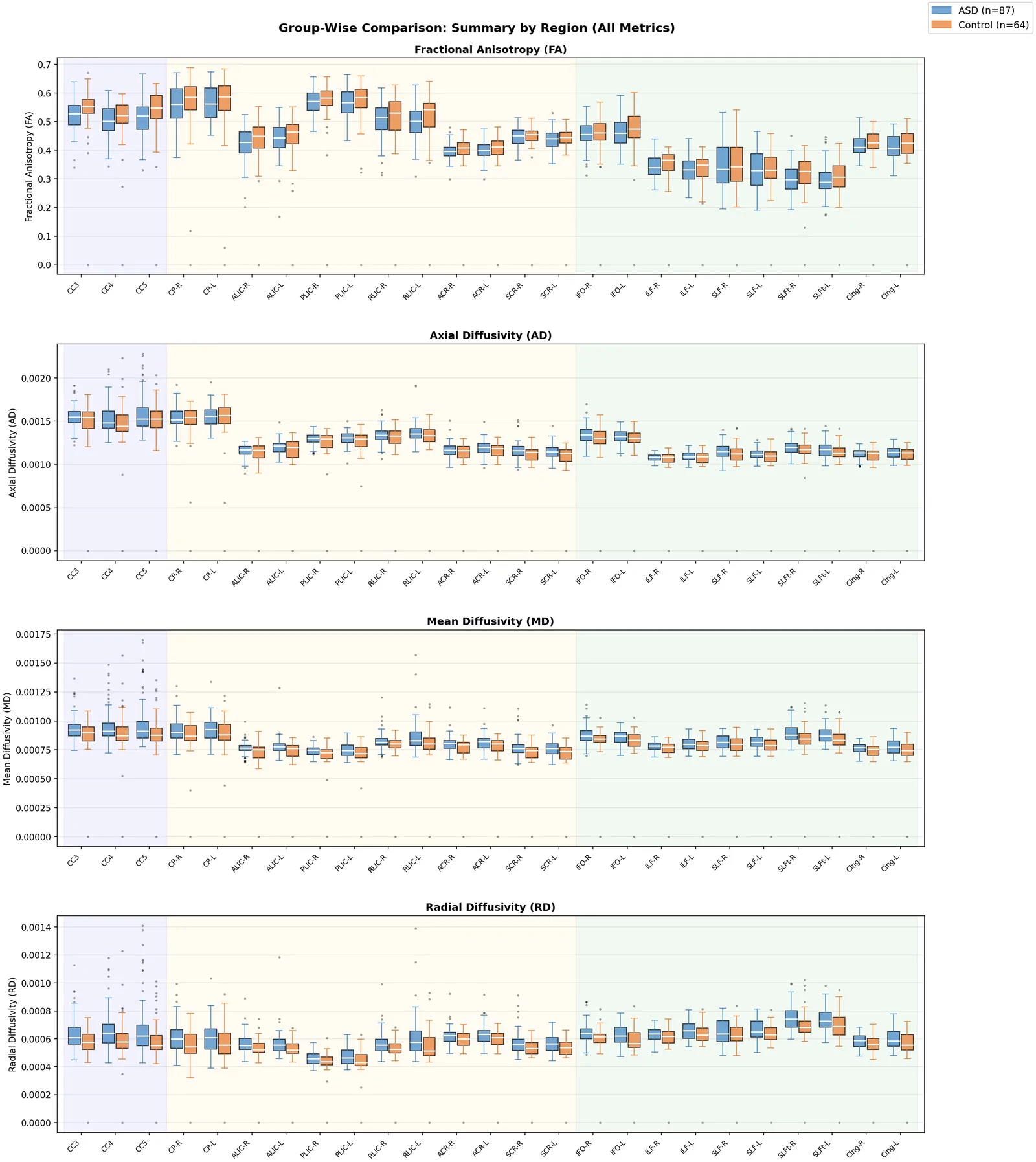
Group-wise comparison: summary by region (all metrics). Regional distributions and mean ± SD values for four major diffusion tensor imaging metrics (FA, AD, MD, RD) in ASD and control cohorts across 25 key white matter tracts. Distinct intergroup differences are evident in select regions, highlighting potential biomarkers and anatomical loci relevant to autism spectrum disorder.

**Figure 4:**
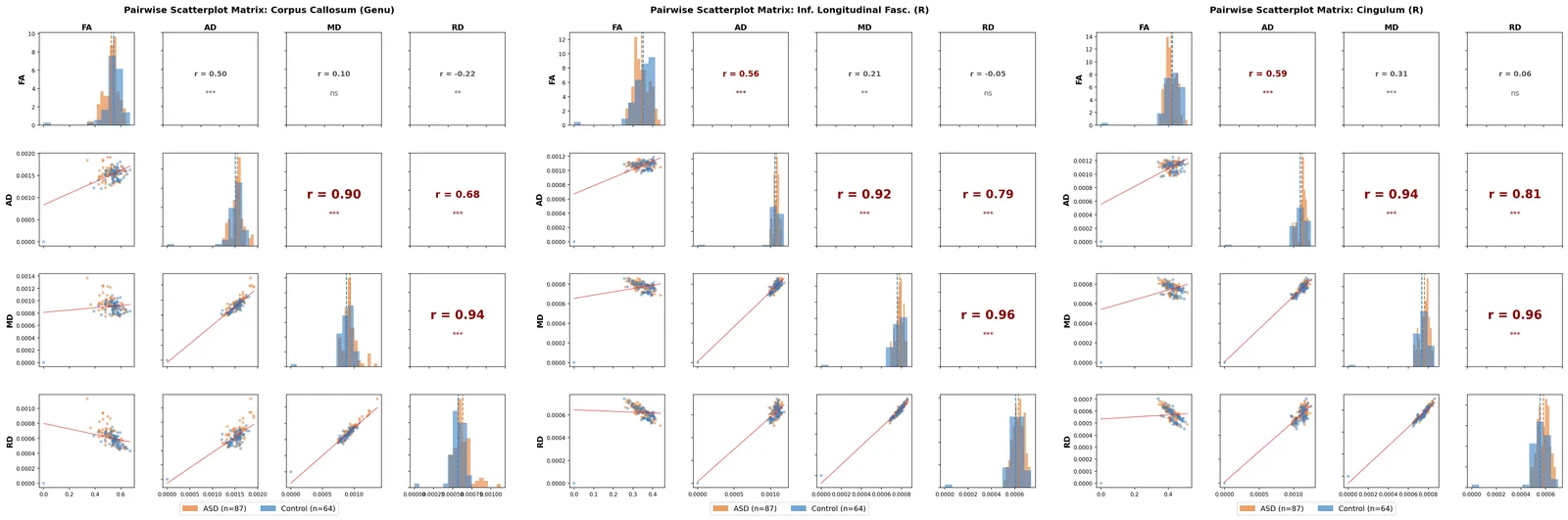
Multivariate pairwise scatterplot matrix: selected regions: [**(A)** corpus callosum genu, **(B)** inf. longitudinal fasc. R, c, cingulum R]. Pairwise scatterplots of four DTI metrics (FA, AD, MD, RD) in corpus callosum, inferior longitudinal fasciculus, and cingulum for ASD and control subjects reveal multiparametric distribution patterns, group-level separation, and nonlinear relationships. Diagonal panels show summary statistics, facilitating identification of outlier and biomarker patterns across diagnostic groups. A control subject with void data is represented as an outlier. ** : p<0.05; *** : p<0.001; n.s, not statistically significant.

**Figure 5:**
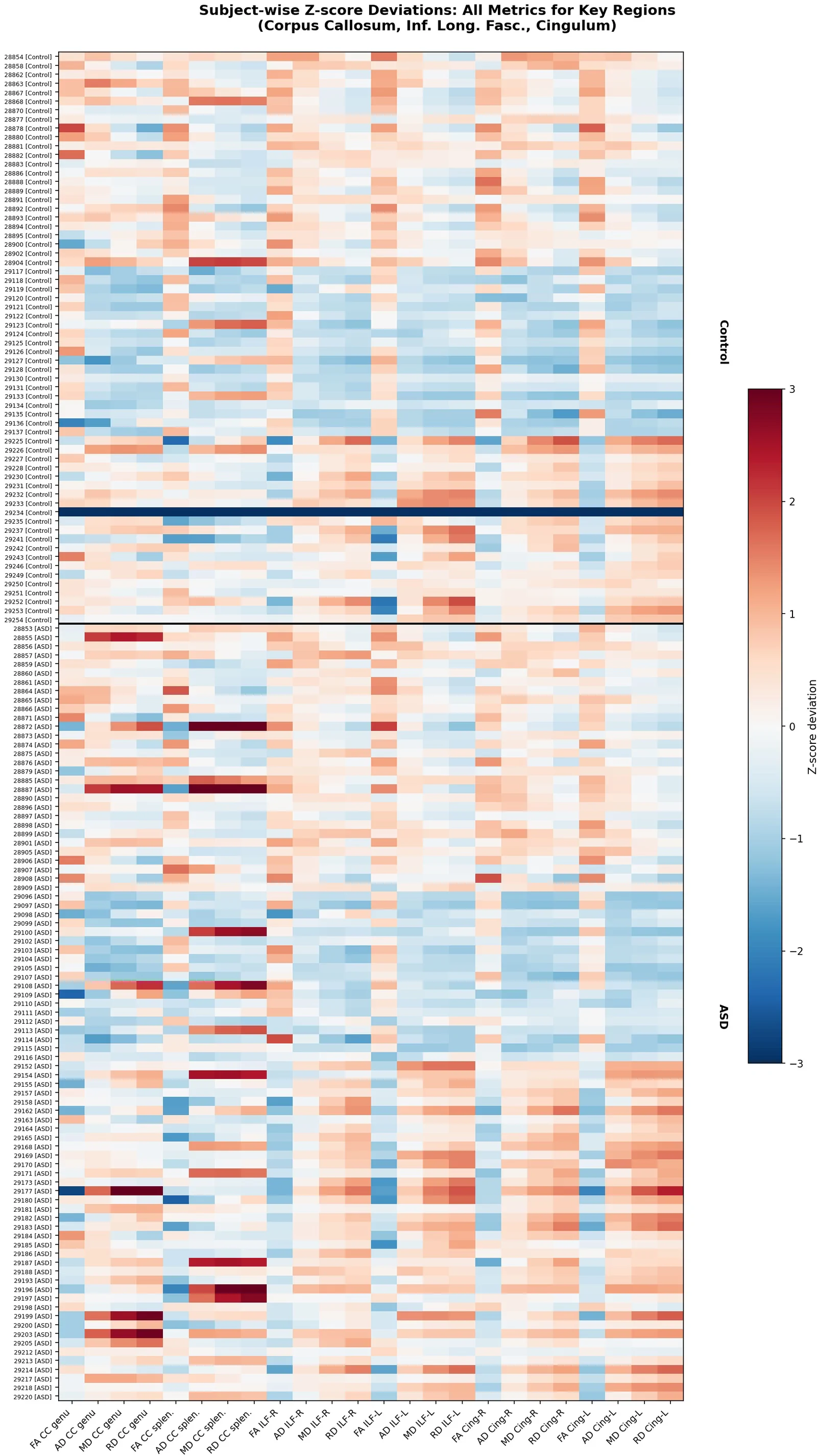
Subject-wise *Z*-score deviations for DTI metrics across key regions. Heatmap visualizing subject-wise *Z*-score deviations for DTI metrics (FA, AD, MD, RD) across six key white matter regions in ASD and control cohorts. Color-coded deviations from cohort means reveal inter-individual and tract-specific variability, with both positive and negative *Z*-scores indicating subjects above or below typical diffusion values for each region-metric combination. The line with zero values in the graph corresponds to the void measurements obtained from processing the images from the subject with image_id = 29234, which were retained in the dataset for transparency after the inclusion/exclusion criteria were applied (Section 2.1). The full version of this figure (i.e., subject-wise *Z*-score deviations for DTI metrics across all regions) is part of the **Supplementary Material**.

This section provides a structured summary of the main DTI patterns present in the dataset, with a focus on tract-level variability, group-wise differences, and cross-metric associations relevant to ASD and neurotypical control cohorts. Visualization of the data demonstrates that FA, MD, AD, and RD metrics capture regional and individual heterogeneity in white matter microstructure across selected anatomical tracts (**Figures 1**–**5**). Consistent group-wide trends include lower FA and higher diffusivity indices (MD, RD, AD) in ASD versus control participants, with the magnitude and direction of differences varying by anatomical region, particularly within the corpus callosum and association fibers. Statistical comparisons and effect size annotations highlight regions characterized by the largest group differences—most notably, corpus callosum subregions exhibit a pronounced reduction in FA among ASD subjects (**Figure 1**). Correlation analyses further indicate strong co-dependence among diffusivity-based metrics, alongside an inverse relationship between anisotropy and diffusivity within select cohorts. Comprehensive visualizations reveal that although group-level distinctions are apparent in key regions and metrics, substantial interindividual and spatial variability persists across the sample, resulting in continuous and overlapping distributions rather than discrete group separations. Data completeness, outlier patterns, and multimetric interactions are also systematically visualized to support transparency and facilitate robust reuse of the dataset for machine learning, biomarker discovery, and reproducibility-driven neuroimaging analyses.

Across all three sampled regions of the corpus callosum (R3, R4, R5), the FA of ASD participants is consistently lower than that of controls, as indicated by negative Cohen’s d values (ranging from − 0.14 to − 0.32), yet the group difference is modest and distributions are nearly overlapping, with all values remaining well above the clinical threshold for abnormally low FA. For AD, MD, and RD, ASD participants exhibit higher metric values compared to controls in each region, with positive Cohen’s d effect sizes that increase from anterior to posterior regions and are most pronounced for RD (Cohen’s *d* up to 0.53). The distributional spread of all metrics in both groups is similar, with wide inter-individual variability and substantial overlap between ASD and control distributions. The most visibly distinct group differences appear in the RD and MD panels, with a separation favoring elevated diffusivity metrics in ASD, although the difference between medians remains less than the full interquartile range. These findings suggest subtle but consistent differences in corpus callosum microstructure between groups, with spatially varying magnitude and direction across regions and DTI metrics. No evidence is observed for marked separation or extreme outliers in either group, and all group mean values fall within physiologically expected ranges.

FA values exhibit substantial regional variability across major white matter tracts, with distinct differences observed between commissural, projection, and association systems. Commissural tracts (corpus callosum regions) show distributions skewed toward higher FA values, indicating greater white matter coherence compared to other systems. Projection tracts present intermediate FA distributions, generally peaking at lower values relative to commissural regions but maintaining moderate anisotropy. Association tracts, including cingulum, external capsule, and longitudinal fasciculi, display the lowest FA distributions, with broader variance and more pronounced leftward tails, suggesting reduced microstructural organization or increased heterogeneity. Across all regions, the presence of outliers and tails at both extremes highlights inter-subject variability. Several regions demonstrate unimodal, sharply peaked distributions, while others are more platykurtic or exhibit mild bimodality. Overall, these patterns reveal that high FA is most consistently observed in commissural tracts, with projection and association tracts showing progressively lower and more dispersed FA values, which may reflect underlying anatomical and clinical differences in white matter microstructure.

Across several white matter regions, the ASD group exhibits visibly lower FA compared to controls, particularly in commissural regions (CC4, CC5), cingulum segments (CG35–CG38), and projection fibers (PD15, PD16, IC19, IC20), with elevated dispersion in some regions. For MD and RD, ASD subjects tend to display higher values relative to controls in many regions, with the most prominent group differences apparent in cingulum (CG36–CG38), external capsule (EC33, EC34), and select projection/association tracts (CR23–CR26, SLF41, SLF42). AD differences are less marked, with group means and spread largely overlapping in most regions. Inter-group divergence in both central tendency and dispersion is most pronounced for FA, MD, and RD in frontal and limbic-associated tracts, supporting their sensitivity to ASD-related microstructural alterations. Some regions exhibit substantial overlap in distribution, indicating limited discriminative value at the group level for those tracts (**Figures 2**, **3**).

The scatterplot matrix reveals multivariate relationships among FA, AD, MD, and RD across three white matter regions (corpus callosum, inferior longitudinal fasciculus, and cingulum) for ASD and control cohorts. Across metrics and regions, distributions are continuous and moderately dispersed, with no evidence of bimodality or clustering that strictly separates diagnostic groups (**Figure 4**). Diagonal panels indicate substantial interindividual variability for most features, and summary statistics confirm that ASD subjects on average exhibit lower FA and higher MD and RD in certain regions, consistent with prior reports of reduced microstructural integrity. Off-diagonal scatterplots demonstrate strong linear correlations between MD and RD within each region, as well as between AD and MD, reflecting the mathematical and biophysical dependencies among tensor-derived metrics. Some panels show a high degree of point overlap between groups, though subtle differences in central tendency are visible in metrics such as FA-CC and MD-Cing. There is no clear pattern of extreme outlier formation specific to ASD or control groups, supporting the presence of continuous, overlapping biomarker distributions rather than discrete diagnostic signatures. No pronounced nonlinear associations are observed among the plotted metrics. Overall, the visualization highlights the joint distributional structure and group-level trends while confirming substantial multivariate overlap between ASD and control subjects.

The heatmap reveals substantial heterogeneity in *Z*-score deviations for DTI-derived features across subjects, with both ASD and control cohorts displaying variable patterns of deviation from cohort means. There is evidence of feature blocks and subject rows exhibiting both marked negative and positive deviations, suggesting interindividual and tract-specific variability. No gross monotonic bias or batch effect is visually predominant across cohorts, although some metrics and regions manifest recurrent high or low *Z*-scores across multiple individuals, meriting targeted review for protocol-driven or biological sources of variation (**Figure 5**).

**Figure 6:**
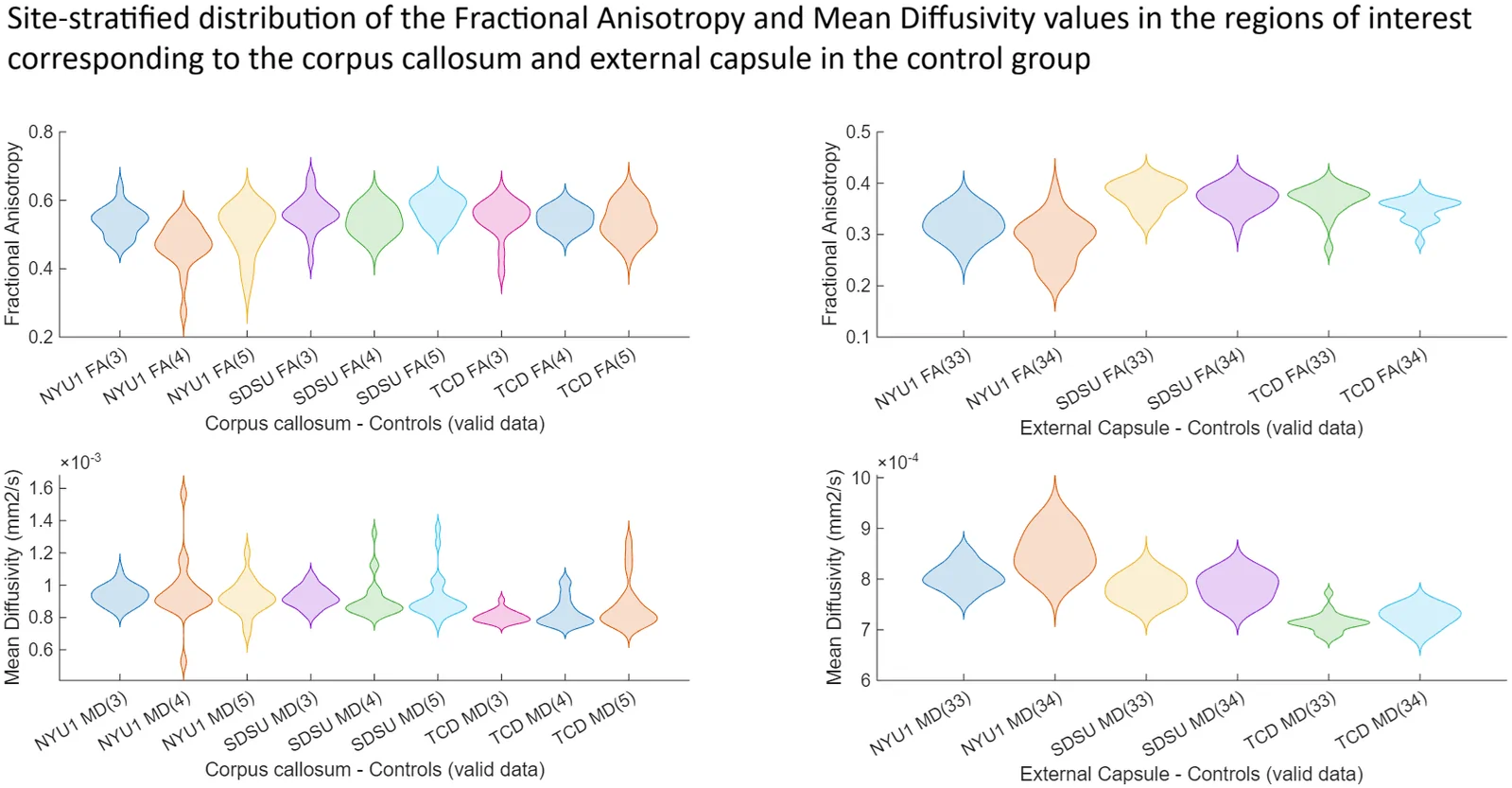
Violin plots showing the distribution of fractional anisotropy (FA) and mean diffusivity (MD) values in the ICBM-DTI-81 regions corresponding to the corpus callosum and external capsule in the control group (subject with void data excluded).

Analysis of the distribution of the data stratified by site shows differences between sites in the average values of FA, MD, AD, and RD in some ICBM-DTI-81 regions (i.e., not all). **Figure 6** shows FA and MD values for the regions corresponding to the corpus callosum and external capsule for controls after excluding the data from the subject for whom the image processing pipeline yielded void values. As residual between-site differences are present, the shifts observed between the means of the distributions per site may be artefactual. The code that generated **Figure 6** is provided as **Supplementary Material** to facilitate exploration of other regions for both control and ASD groups. Further harmonization of the data per site and image acquisition protocol using two harmonization methods is also provided as part of the supplementary material.

## 4 Discussion

### 4.1 Value and applications of the dataset

This dataset offers a structured resource for investigating white matter microstructure in neurodevelopmental and neuropsychiatric research, with a particular emphasis on ASD and matched control populations. The collection provides regionally averaged measures of FA, AD, MD, and RD across defined anatomical regions in the JHU ICBM-DTI-81 atlas for both ASD and control groups. The integration of spatially registered, voxel-averaged tensor metrics enables detailed regional comparisons and supports studies assessing alterations in brain connectivity and white matter properties.

The dataset supports a range of potential research questions, including the characterization of regional diffusion properties in ASD relative to typical development, exploratory analyses of structure–function relationships, and the evaluation of group differences using interpretable summary statistics. It is well-positioned for analyses examining correlations between diffusion metrics and other phenotypic or demographic variables, the development of machine learning models aimed at classification or regression tasks, or benchmarking algorithms for automated region-based tractometry.

Compared to existing datasets, this collection distinguishes itself by providing uniformly processed, harmonized, region-based diffusion parameters derived from a standardized neuroimaging atlas derived from a publicly available database, covering several white matter tracts relevant to neurodevelopmental conditions. It extends the utility of previous studies by structuring the data for reproducible, region-wise quantitative analyses, and by supplying well-documented metadata, variable descriptions, and summary statistics. The interoperability with common neuroimaging frameworks and adherence to FAIR^2^ standards further enhance its applicability for both hypothesis-driven and exploratory research within the neuroimaging community. It does not contain any duplicate information from the primary database, namely ABIDE II, nor from any of the datasets or projects already derived from it (i.e., PCP, MLLE, LADDER). This dataset does not substitute them but rather complements and enhances these data initiatives to facilitate research in ASD. By using the same image identifications as the primary database, it facilitates data linkage between repositories, and its generation using state-of-the-art, well-established DTI processing methods ensures full compatibility with other datasets generated under current practices, making it suitable for incorporation into artificial intelligence-powered frameworks.

By providing the raw data obtained from a state-of-the-art image processing pipeline, together with the code to harmonize the data considering differences in acquisition protocol and the harmonized data as **Supplementary Materials**, we offer users options to suit a variety of purposes and preferences. Some researchers may prefer to use the raw data and consider the effect of intersite differences as a covariate in their statistical models rather than using, for example, the *Z*-score-harmonized data (or vice versa), while AI developers may need to use the raw data for their AI model to learn heterogeneity in the input data.

### 4.2 Limitations of the dataset

This dataset captures regionally aggregated measures of fractional anisotropy, axial diffusivity, mean diffusivity, and radial diffusivity across several labeled white matter regions, derived from preprocessed DTI data for both ASD and control groups. While this structure supports comparative and correlational analyses in neurodevelopmental studies, several constraints warrant consideration. The dataset is limited to the selected JHU ICBM-DTI-81 atlas regions, which may restrict investigation to macroanatomical structures and exclude microstructural or nonatlased regions. The absence of data regarding confounding variables (such as age, sex, medication status, or scanner site) may limit adjustment for potential sources of variance unrelated to diagnostic status. Certain variables exhibit zero minimum values; this could represent either true physical boundaries in the presence of contaminating signals, numeric thresholding, undetected q-form errors, or unrecognized artifacts, and may bias statistical analyses if not carefully screened. The aggregation of voxelwise data into regional means, while facilitating cross-subject comparison, necessarily reduces within-region spatial heterogeneity and may attenuate detection of focal abnormalities. The dataset does not include imaging modalities beyond DTI, nor does it contain raw imaging or quality control information, limiting the scope for multimodal or technical validation analyses. There are no provided data elements indicating intrarater reliability, scan–rescan consistency, or extended demographic annotations, which may pose challenges for reproducibility and generalizability. The strong correlation among AD, MD, and RD (*r* ≈ 0.90–0.96), resulting from the formula MD = (AD + 2·RD)/3, reduces the effective number of independent features to 50 (i.e., half of the original number of features)—a factor to consider in analyses involving this dataset (see 2). Finally, the data are not structured according to standard neuroimaging formats such as BIDS, which may limit immediate interoperability with established pipelines or initiatives. These factors should be carefully weighed when determining the suitability of the dataset for specific analytic or modeling purposes.

### 4.3 Opportunities for future research

This dataset provides regionally aggregated DTI metrics—fractional anisotropy, axial diffusivity, mean diffusivity, and radial diffusivity—across various neuroanatomical regions for both ASD and control cohorts. Its structure enables reproducible comparison of group-level microstructural characteristics in accordance with contemporary data stewardship principles. To further increase utility, future iterations may benefit from (1) the inclusion of additional anatomical regions or higher spatial resolution; (2) the harmonization of data collection and preprocessing protocols to minimize site and batch effects; (3) the enrichment of subject-level metadata, including demographic, clinical, or behavioral variables; and (4) the adherence to domain standards such as BIDS for enhanced data interoperability. Integration with multimodal neuroimaging data (e.g., structural MRI, functional imaging) would support multimodal analyses and potential cross-modality biomarker research. Expansion to longitudinal measurements could facilitate studies of temporal dynamics. Comprehensive provenance and bias documentation would further support responsible reuse, particularly for AI-based investigations. Overall, the dataset is positioned to contribute as a benchmark resource for quantitative neuroimaging studies, with clear avenues for structured improvements aligned with advances in open data sharing, AI readiness, and responsible research practices.

## 5 Conclusions

The final dataset provides a carefully structured resource for the quantitative characterization of white matter microstructural features in individuals with ASD and matched control participants, as derived from the ABIDE II neuroimaging repository. The dataset offers explicitly documented regional DTI metrics—FA, AD, MD, and RD—systematically mapped to anatomically defined white matter regions of interest based on the JHU ICBM-DTI-81 atlas. Each variable’s provenance is transparently defined, linking computational features to their corresponding image processing and feature extraction steps as annotated in the methods and data dictionary, ensuring traceability and reproducibility.

The data organization adheres to the FAIR^2^ framework, prioritizing findability, accessibility, interoperability, reusability, and responsible AI readiness. The accompanying metadata and schema annotations facilitate not only human interpretability but also support machine-actionable workflows relevant for advanced analytics, statistical modeling, and algorithmic development. Quality control, internal consistency, and methodological transparency are maintained throughout the dataset lifecycle, establishing a robust foundation for downstream use.

By documenting all data processing, feature definitions, and structural logic, this resource supports a broad spectrum of secondary research, including exploratory, predictive, and bias mitigation analyses in neuroimaging and computational neuroscience. The dataset’s integrity, provenance, and formal structuring collectively empower reproducible, transparent, and ethically informed investigations into the structural connectivity features relevant to ASD.

## Data Availability

The dataset is FAIR^2^-certified and publicly available under an open license permitting unrestricted reuse with appropriate attribution. Access is provided through two coordinated components: an interactive FAIR^2^ Data Portal for visual exploration and quality assessment, and a downloadable FAIR^2^ Data Package containing harmonized data products, structured metadata, and detailed documentation. The FAIR^2^-compliant Data Package, which includes the interactive Data Portal, is published independently through the FAIR^2^ infrastructure with its own DOI (https://www.doi.org/10.71728/senscience.dbrh-5zc8) and is indexed in DataCite to support discoverability, reproducibility, and downstream integration.

## References

[1] Di Martino A O’Connor D Chen B Alaerts K Anderson JS Assaf M . Enhancing studies of the connectome in autism using the autism brain imaging data exchange II. Sci Data. (2017) 4:1–15. doi: 10.1038/sdata.2017.10 28291247 PMC5349246

[2] Cardenas-Hernandez NA Perez-Diaz M García-Ramó KB Valdés Hernández MDC . Autism spectrum disorder detection using diffusion tensor imaging and machine learning. PloS Digit Health. (2025) 4:e0001155. doi: 10.1371/journal.pdig.0001155 41433275 PMC12725754

[3] Hua K Zhang J Wakana S Jiang H Li X Reich DS . Tract probability maps in stereotaxic spaces: analyses of white matter anatomy and tract-specific quantification. Neuroimage. (2008) 39:336–47. doi: 10.1016/j.neuroimage.2007.07.053 17931890 PMC2724595

[4] Gorgolewski KJ Auer T Calhoun VD Craddock RC Duff EP Flandin G . The brain imaging data structure, a format for organizing and describing outputs of neuroimaging experiments. Sci Data. (2016) 3:160044. doi: 10.1038/sdata.2016.44 27326542 PMC4978148

[5] Tournier J-D Smith R Raffelt D Tabbara R Dhollander T Pietsch M . MRtrix3: A fast, flexible and open software framework for medical image processing and visualisation. Neuroimage. (2019) 202:116137. doi: 10.1016/j.neuroimage.2019.116137 31473352

[6] Schilling KG Blaber J Hansen C Cai L Rogers B Anderson AW . Distortion correction of diffusion weighted MRI without reverse phase-encoding scans or field-maps. PloS One. (2020) 15:e0236418. doi: 10.1371/journal.pone.0236418 32735601 PMC7394453

[7] Jenkinson M Beckmann CF Behrens TEJ Woolrich MW Smith SM . FSL. Neuroimage. (2012) 62:782–90. doi: 10.1016/j.neuroimage.2011.09.015 21979382

